# Supplementary material for: Learning curve and surgical outcome of robotic assisted colorectal surgery with ERAS program
Source: Sci Rep. 2022 Nov 29;12:20566. doi: 10.1038/s41598-022-24665-w (PMC9709162; doi:10.1038/s41598-022-24665-w)
Supplement: Supplementary file 2 — Supplementary Information 2. [file 41598_2022_24665_MOESM2_ESM.zip › RAL Raw data-Ñ[▒K/087/OP N_ñw╝╨░O▒Kñσ.pdf]

W52 / W52

臺中榮民總醫院
手術室護理記錄

[redacted]

基本資料

- 室別： 開刀房-17室，手術科別： CRS，手術類別： 常規，日期： 2021/05/29
- 進入時間： 0805 ； 麻醉開始時間： 0825 ； Time Out時間： 0930 ； 劃刀時間： 0931 ； 手術結束時間： 1315 送出時間： 1345 ；
- 麻醉方式： GE

  診斷： Malignant tumor of sigmoid colon 20CM FAV

  手術名稱： Robot Assisted Colectomy(L)

  手術局部用藥： nil

  術前預防性抗生素：
  - 抗生素名稱一： Veterin 1GM IVD/由麻醉護理師給予
  - 時間： 0927
  - 上次簽章: 陳盈妤

  ，追加預防性抗生素：
- 手術醫師： 蔣鋒帆，楊曙榮
- 麻醉醫師： VS 謝昀叡
-
  - 刷手護士A ： 羅媗鈴，時間： 0805，刷手護士B ： 陳盈妤，時間： 0805，接替刷手一： 羅媗鈴/黃鈺雯，時間： 0900，接替刷手二： 陳盈妤，時間： 1230
  - 流動護士A ： 陳麗君，時間： 0805，接替流動一： 陳盈妤，時間： 0900，接替流動二： 林貝臻，時間： 1200
- 更多接替及流動護士： 接替流動三： 羅媗鈴，時間： 1315

皮膚狀況

A-V Shunt： 無

術前： 完整

記錄者： 上次簽章: 陳麗君

術後： 完整

記錄者： 上次簽章: 林貝臻

手術準備

手術臥式 單一手術臥式

-
  - 手術臥式： 截石臥式，NIL
  - 使用附件： 溫毯 40 ℃，其它： 海綿軟墊x4
  - 皮膚準備： 10%Aq Betadine，Hibiscrub，2% Chlorhexidine，其它： NIL
  - 電燒： 使用電燒: 電極片位置： 其它： NIL，右大腿，其它： NIL，記錄者： 上次簽章: 陳盈妤

止血帶T： 無

植入物

- 植入物名稱/號碼/數量/部位： COVIDIEN GIA 80-3.8mm X 2/D-colon

，記錄者： 上次簽章: 林貝臻

標本送檢

- 細菌培養 無
- 細胞學 無
- 冰凍切片 無
- 病理標本

  組織名稱一： D-colon

  ，

  組織名稱二： Omentum
- 截肢 無

標本交付 標本： 2 袋，標本： 0 盒，標本： 0 瓶，細胞學： 0 管，Culture Tube： 0 管，被交付醫師： CR 楊曙

榮，

- 記錄者： 羅媗鈴
- 時間： 1345

傷口術後數料

傷口數料： 傷口數料： 無

- 請於圖上標示部位：

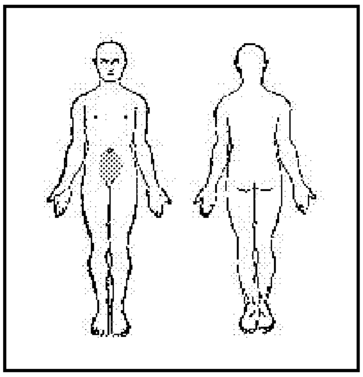

- 傷口數料： Trocar:mepore

導管

導管	天數	導管資訊	插入日期	插入時間	更換日期	拔除日期	拔除時間	拔除原因	預計拔除/更換	預計拔除/更換日期
Epidural PCA(1)	1	部位:硬脊膜下;	2021/05/29	0820						
Double Lumen CVC(1)	1	號碼:8Fr. ; 部位:; 象限:RU; 註記:fix 15cm;	2021/05/29	0855						
A-line (1)	1	號碼:20G ; 部位:左手;	2021/05/29	0845						
Foley(1)	1	號碼:16 Fr.; 部位:膀胱; 註記:Balloon 10ml;	2021/05/29	0915						

用物計數

- 手術中出血量： minimal c.c.
- 手術中尿量： 300 c.c.
-
    - 其它： 無
    - NIL

用物計數： 多切口同時縫合

- 1.器械： 正確，2.縫針： 正確，3.刀片： 正確，4.紗布： 正確，5.腹部墊： 未使用，6.紗球： 未使用，7.PEANUTS ： 未使用，8.紗條： 未使用，9.絲帶： 正確，10.針頭： 未使用，11.Vessel loop： 未使用，12.Boots： 未使用，13.Jelco： 未使用，14.棉條： 未使用，
    - 第一次：
        - 流動護士：

,

    - 第二次：
        - 流動護士：

,

    - 第三次：
        - 流動護士：

備註

備註： Davinci XI 30度scope:NO.1

- 記錄者： 上次簽章: 陳盈妤
- 時間： 0930

填寫護理人員 羅媗鈴

正式摘要

W52 / W52

doctorRank:B
